# Improving emergency and admission care in low-resource, high mortality hospital settings—not as easy as A, B and C

**DOI:** 10.1093/heapol/czab128

**Published:** 2021-10-22

**Authors:** Mike English

**Affiliations:** KEMRI-Wellcome Trust, Nairobi, Kenya; Health Systems Collaborative, Nuffield Department of Medicine, University of Oxford, Oxford, UK

**Keywords:** Implementation, quality of care, study design, complexity

**Key messages**

- Strategies to provide emergency care training and promote use of clinical guidelines targeting common severe illness in newborns and children are available but there remain few studies testing the consequences of introducing these through multifaceted interventions.
- Unlike studies that compare two quite specific treatments (e.g. drug A vs drug B) interventions that target improvement in delivery of emergency care and multiple guidelines require many changes in the organization and behaviour of multiple health workers (e.g. so that a team follows the key A, B and C steps of an emergency protocol and then continues to offer correct care over hours or days).
- Some approaches to evaluating such multi-faceted interventions try and specify the complicated but logical sequence of changes or steps in care that need to be achieved so that patient outcomes including mortality are improved. These approaches may also recognize the complicated set of contextual factors that may influence implementation itself and patient outcomes. In principle, good study designs and analytic models might then account for all such influential factors to provide good evidence of effect for decision makers.
- There are concerns that even very carefully designed studies that treat health care delivery as a complicated problem, especially those focused on organizations as the unit of study, may not in the end provide the evidence needed. Instead, we need study designs and research platforms that deal with complex systems.

The number and quality of clinical trials examining specific alternative disease treatments (A vs B) in low-resource settings (LRSs) has increased because of dedicated funding streams. Rigorous evaluations of innovations or improvements in service delivery such as that now reported by Hategaka *et al* are far fewer (Hategeka *et al.*, 2021). In this example the intervention (an ETAT+ package (Irimu *et al.*, 2008)) aimed to promote use of multiple recommended practices to improve quality of hospital care in Rwanda. These included changing the way individual health-workers and teams organize their practice (e.g. care guided by the A, B and C’s for triage and resuscitation) and correctly identifying and treating children and newborns with specific conditions (e.g. diagnosis and management of severe dehydration or sepsis) (Hategeka *et al.*, 2021).

In many ways improving service delivery seems self-evidently a good thing. Who, anywhere in the world, would not wish their sick child or newborn to have access to a hospital where health workers have the systems and skills to handle serious illness effectively? So, what should we make of a controlled interrupted time-series study of a multi-faceted intervention to achieve this in Rwanda that had no statistically significant impact on all-cause neonatal and paediatric hospital mortality, although a possible impact on the case-fatality of targeted neonatal conditions? (Hategeka *et al.*, 2021). For some, the answer will be simple. Strictly, this study provides insufficient evidence that the intervention works well enough, so why use it? For others, providing evidence on what improves hospital care and outcomes is more complicated. For yet others, the answer is well, complex.

Making things complicated are the number and range of issues that make designing, conducting and interpreting multi-faceted interventions targeting clinical care in hospitals problematic. I reflect briefly on just three; how do interventions work, achieving control in comparative studies and choosing the right measures of success. Firstly, we ought to be able to articulate how our intervention should achieve our desired outcomes. Logic models, theories of change and directed acyclic graphs may help and may be supported by other approaches for understanding implementation (Michie *et al.*, 2011; Powell *et al.*, 2015; De Silva *et al.*, 2014). This conceptual thinking should identify key intermediary clinical practice changes needed to produce mortality effects if this is their goal. Ideally prospectively developed models then drive specific data collection on important intermediary effects. For example, in studies testing interventions to improve triage, emergency and immediate admission care impacts on mortality seem predicated on improving the speed and accuracy with which sick children are seen, assessed and managed. So, do such intermediary outcomes change?

Here, we need to distinguish evaluating intermediate effects from fidelity of intervention delivery (Moore *et al.*, 2015). The latter is primarily concerned with whether or how well what is planned is done. Failure to implement may explain why both key intermediary effects and final outcomes are not achieved. Conversely, if implementation goes as planned but key intermediary effects are not seen how do we interpret any change in our main outcome measure? Examining intermediary effects early may even call into question the value of proceeding to a multi-year mortality endpoint and limit research waste. Unfortunately, an overarching challenge with service delivery interventions and models of them are they can become hugely complicated. A recent typology included over 70 distinct implementation components that might be deployed (Powell *et al.*, 2015). In one Kenyan hospital improvement project, over 20 were deployed simultaneously and evaluating their successful delivery and multiple intermediary effects can become an overwhelming task (English *et al.*, 2017).

Such complicated interventions also pose challenges for ‘controlled’ studies. Hategaka *et al.’s* study design is said to control for ‘differences in characteristics between intervention and control hospitals that remained constant or changed over time’ (Hategeka *et al.*, 2021). When hospitals are the units of intervention the numbers involved in trials are typically few. Conversely, the number of factors that may act as confounders or introduce bias is extremely large, and many may be important but unmeasurable (e.g. clinical leadership) (English *et al.*, 2011). Is it then reasonable to expect that two groups of hospitals are ‘balanced’ with respect to a huge array of largely unknown but influential factors? Furthermore, such factors may vary across place over time. For example, an influential clinical leader is transferred from one hospital to another. How is such a change controlled for other than by assuming that changes ‘balance out’ over time between intervention and control groups. Is this likely when relatively small numbers of organisations are studied? If we cannot verify assumptions of balance how safe are we ever making assumptions of internal validity? (English *et al.*, 2011).

Impacts on mortality to justify intervention in high-mortality settings is often what we desperately want and what funders demand. It typically means improving many intermediary aspects of quality of care as discussed above. However, is mortality a good aggregate measure of quality care? Many would say no because mortality is highly dependent on case-severity and case-mix that vary across place and time (Lilford and Pronovost, 2010). For, example, we have seen three-fold variation in mortality across hospitals in Kenya (Irimu *et al.*, 2021). Adjusting for these factors requires detailed individual patient data which is rarely available. Perhaps more critically mortality is strongly influenced by the cumulative quality and safety of care (or its absence) prior to and over days or even weeks of admission. So how reasonable is it to expect that improving important but time-bound aspects of care will impact mortality when whole systems are weak? Some trials of service delivery interventions have demonstrated effects on hospital mortality in LRS, but these have employed randomization at the individual level and well-resourced, sustained and trial-supported change efforts (Biai *et al.*, 2007; WHO Immediate KMC Study Group, 2021). When interpreting these, the contribution of an often supernumerary ‘trial team’ is frequently ignored as an input. So, if we cannot demonstrate improved mortality are interventions that (only) improve care processes useful? Very small improvements in hospital mortality that are almost impossible to ‘prove’ are associated with intervention may still be highly cost-effective (Barasa *et al.*, 2012). So should interventions that do not impact mortality but that improve adoption of multiple evidence-based therapies or management steps be taken to scale?

We outline above how the challenges of statistically testing A vs B interventions while characterizing and accounting for every complicated aspect of context and intervention delivery may be insuperable. Some now regard pursuit of this elaborate but still reductive approach to miss the point because in high and lower-resource settings we are dealing with Complex (Adaptive) Systems. These defy explanation based on linear cause and effect models and it is beyond the scope of this article to explain these ideas in full (but to introduce the topic see (Greenhalgh and Papoutsi, 2018)). Their importance is what they mean for evaluators of service delivery interventions. Such evaluations are very rarely simple A vs B comparisons. Nor are they even tests of A + B + C intervention packages complicated by factors X, Y and Z (and more), representing measurable variations in context or fidelity. Instead, to develop the field we need strategies for evaluating service delivery interventions that pay attention to complexity and clinical researchers need to partner with and learn from and employ methods often developed by social scientists, economists, engineers and others. Also, critical is ensuring those with knowledge of the context and systems are central to evaluation as ‘insider knowledge’ is key to building understanding. At the same time, we should also examine outcomes valued by policy makers, practitioners and communities who ultimately have the task of sustaining implementation (Gilson *et al.*, 2021). Embedded, multi-disciplinary research linked to collaborative learning platforms with good historical data on context and key outcomes may be especially helpful in this regard (English *et al.*, 2021).

## Data Availability

Data are not presented or used in this commentary.
